# Multi-Phase Design Strategy for Synergistic Strength–Ductility Optimization in V-Ti-Cr-Nb-Mo Refractory High-Entropy Alloys

**DOI:** 10.3390/ma18112479

**Published:** 2025-05-25

**Authors:** Xinwen Liang, Jiahao Zhu, Zhenjiao Tan, Ruikang Chen, Yun Chen, Xiaoma Tao

**Affiliations:** 1 Guangxi Key Laboratory for Relativistic Astrophysics, School of Physical Science and Technology, Guangxi University, Nanning 530004, China; 2212270207@st.gxu.edu.cn (X.L.); 2212270225@st.gxu.edu.cn (J.Z.); 2212270242@st.gxu.edu.cn (Z.T.); 2212270234@st.gxu.edu.cn (R.C.); 2212270231@st.gxu.edu.cn (Y.C.); 2 State Key Laboratory of Featured Metal Materials and Life-Cycle Safety for Composite Structures, School of Physical Science and Technology, Guangxi University, Nanning 530004, China

**Keywords:** refractory high-entropy alloy, multi-phase, microstructure, mechanical properties

## Abstract

Controlling multiple phases by adjusting elemental ratios and applying heat treatments effectively balances the strength and ductility of refractory high-entropy alloys. In this study, five types of V-Ti-Cr-Nb-Mo alloys were designed by varying the contents of V, Ti, and Nb, followed by annealing at 1200 °C for 8 h. The alloys’ crystal structures, microstructure evolution, and mechanical properties were systematically investigated. The V-Ti-Cr-Nb-Mo alloys exhibited a typical dendritic structure with a dual-phase (BCC + HCP) matrix. When the Nb content was maintained at 35 at.% with increasing V content, the volume fraction of the HCP phase increased, and the C14 Laves phase emerged. The as-cast alloy V_15_Ti_30_Cr_5_Nb_35_Mo_15_, with a triple-phase (BCC + HCP + Laves) structure, exhibited excellent mechanical properties, including a compressive strength of 1775 MPa and a ductility of 18.2%. After annealing, the HCP phase coarsened and partially dissolved, the Laves phase precipitation reduced, and while the hardness and strength decreased, the ductility improved significantly. The annealed alloy V_5_Ti_35_Cr_5_Nb_40_Mo_15_, with a dual-phase (BCC + HCP) structure, achieved a ductility of 26.9% under a compressive strength of 1530 MPa. This work demonstrates that multi-phase refractory high-entropy alloys can significantly enhance the strength–ductility synergy, providing an experimental foundation for the compositional design and performance optimization of refractory high-entropy alloys.

## 1. Introduction

The rapidly advancing aerospace industry has a significant demand for high-performance, high-temperature-resistant superalloys. Ni-based superalloys are extensively employed in hot-section aero-engine components due to their outstanding high-temperature properties, including high strength, fatigue resistance, and creep resistance [1,2,3]. However, the practical application temperature of most nickel-based superalloys is limited to approximately 1150–1200 °C [4,5,6]. Therefore, exploring high-performance superalloys within alternative alloy systems, such as Nb-based [7] and Co-based [8], is significant.

Senkov [9,10] was the first to apply the high-entropy alloy concept to develop a series of refractory high-entropy alloys (RHEAs). The NbMoTaW RHEAs, fabricated via arc melting, not only retain a stable single-phase BCC structure at 1400 °C but also exhibit a compressive yield strength of 405 MPa, compressive strength of 600 MPa, and compressive strain of 27% under compression at 1600 °C. Although their high-temperature mechanical properties significantly exceed nickel-based superalloys, their compressive strain at room temperature is limited to only 1.5%. The application of RHEAs is often restricted by room-temperature brittleness resulting from the single-phase BCC structure. A balance between strength and ductility can be achieved through multi-phase design strategies, such as incorporating HCP or Laves phases.

Controlling the proportion of alloying elements is a common approach to achieving a multi-phase microstructure. For example, in CoCrFeNiTi_x_, multi-principal element alloys, σ, Laves, and Eta phases can be formed by adjusting the alloy composition, such as by adding Ti. These newly formed phases significantly enhance the strength and fracture toughness of the alloy, demonstrating that multi-principal element alloys with dual or multi-phase structures can improve the strength–ductility synergy [11]. By adjusting the Ti and Al content in Fe-Cr-Ni-Ti-Al alloys, a Laves phase is formed, resulting in a multi-phase microstructure consisting of disordered BCC A2, ordered BCC B2, and Laves phases, which improves the alloy’s ductility [12]. Studies have demonstrated that high-temperature heat treatments, such as annealing and aging, can effectively regulate the phase composition of RHEAs, promote the precipitation and stabilization of specific phases, and optimize their microstructure and mechanical properties. For example, after the ZrNbTaHf_0.2_Cr_x_ alloy undergoes heat treatment at 1400 °C for 24 h, the phase fraction of the C15 Laves phase significantly increases, accompanied by enhanced hardness and yield strength [13]. ZrNbTaHf_x_ alloys form an HCP phase after annealing at 1500 °C for 24 h, considerably strengthening the alloy’s hardness [14]. In addition, mechanical treatments, such as high-pressure torsion, mechanical alloying, and cold rolling, can also effectively modify the phase composition of RHEAs by regulating element distribution, promoting grain refinement, increasing dislocation density, and influencing phase transformation behavior [15,16,17].

Previous research on V-Ti-Cr-Nb-Mo RHEAs mainly concentrates on the impact of changing monatomic or binary element ratios on the phase structure [18,19,20,21]. In contrast, the synergistic mechanisms between V, Ti, Nb, Cr, and Mo and phase evolution under heat treatment are poorly understood. Titanium, which has an HCP structure at room temperature, changes to a BCC structure at high temperatures [16], and adding Ti to Cr-based alloys can effectively encourage the formation of the Laves phase [22]. Nb and V have stable BCC crystal structures at both room temperature and elevated temperatures, and the stability of the BCC phase can be controlled by altering their concentrations, which indirectly aids in the precipitation of other metastable phases. Accordingly, five groups of V-Ti-Cr-Nb-Mo RHEAs were designed by varying the concentrations of V, Ti, and Nb and heat-treated. The microstructures of the as-cast and annealed RHEAs were characterized using X-ray diffraction (XRD), electron probe microanalysis (EPMA), and transmission electron microscopy (TEM). The mechanical properties of the V-Ti-Cr-Nb-Mo RHEAs were evaluated, and their fracture morphologies were subsequently analyzed. The interrelationships among composition, phase structure, and mechanical properties were systematically investigated.

## 2. Materials and Methods

Five alloys were prepared by a non-self-consuming vacuum arc melting furnace using high-purity (=99.95%) V, Ti, Cr, Nb, and Mo particles as raw materials. The compositions of the five V-Ti-Cr-Nb-Mo RHEAs designed in this study are summarized in Table 1. The copper crucible is externally cooled with a water-cooled refrigeration condensing unit set to 18 °C. Before melting, low melting point and volatile elements were positioned at the bottom of the copper crucible to minimize loss during processing. The melting process started by setting the current to 20 A and pressing the arc-striking button. The current was then gradually increased to 230 A. To ensure compositional homogeneity, each ingot was re-melted 11 times under an argon atmosphere, remaining in the liquid state for approximately 3 min during each cycle. The ingots had a diameter of approximately 30 mm. After melting, the current was reduced, and the arc was extinguished. The prepared alloys were annealed at 1473 K for 8 h and quenched in water. During annealing, the samples were sealed in a tubular furnace, which was evacuated and backfilled with high-purity argon to prevent oxidation. A wire-cut electric discharge machine obtains the central part of the button ingot.

The crystal structure was analyzed using an X-ray diffractometer (XRD, Rigaku, Tokyo, Japan, Smart Lab, 3 kW), operated at 40 kV and 30 mA with Cu Kα radiation (λ = 1.5406 Å). XRD analysis was performed at a speed of 6°/min over the scanning angle (2θ) range of 20° to 100°. The microstructure was examined using scanning electron microscopy (SEM, Zeiss, Jena, Germany, Sigma 500) equipped with backscatter electron (BSE) detectors. Before observation, the square surface of the sample was polished using conventional metallurgical techniques to achieve a smooth and flat finish. The metal blocks were subsequently cleaned in an ultrasonic cleaner to remove dust and grease and then dried. A transmission electron microscopy (TEM, FEI, Hillsboro, OR, USA, Tecnai G2 F20) was operated at 200 kV to analyze the microstructure further. The distribution of alloying elements was quantitatively analyzed using electron probe microanalysis (EPMA, JEOL, Tokyo, Japan, JXA-8230) with wavelength dispersive spectroscopy (WDS). The density of RHEAs was determined using Archimedes’ method with an analytical balance (Mettler-Toledo Swiss, Greifensee, Switzerland, AG285) at 20 °C. The Vickers hardness test was carried out by a Vickers Hardness Tester (Qness, Golling an der Salzach, Austria, Q30A). A load of 1000 g was applied and maintained for 10 s, and the average value of 7 random positions was taken to determine the hardness. The compression test was performed on the cuboid specimens with dimensions of 5 × 5 × 10 mm using an INSTRON-8801 machine (Norwood, MA, USA) with an initial strain rate of 1.0 × 10^−3^ s^−1^ at room temperature.

## 3. Results and Discussions

### 3.1. Crystal Structures and Microstructure Evolution

**Figure 1:**
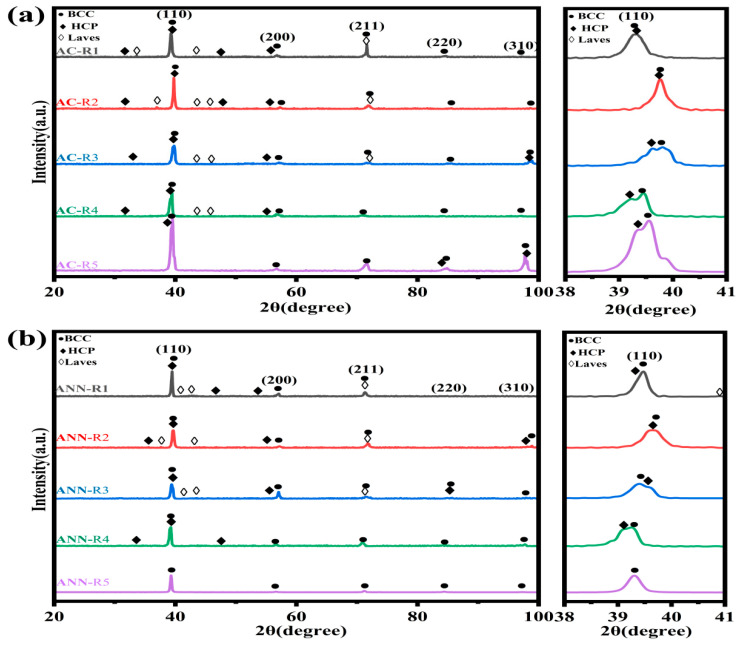
XRD patterns of the V-Ti-Cr-Nb-Mo RHEAs: (**a**) as-cast state; (**b**) annealed state.

Figure 1 presents XRD patterns of as-cast and annealed V-Ti-Cr-Nb-Mo RHEAs. All samples (AC-R1 through AC-R5) were based on the (BCC + HCP) phases, as shown in Figure 1a. The volume fraction of the HCP phase increases with increasing V content while maintaining the Nb content at 35 at.%, and the C14 Laves phase emerges. This suggests that interactions between V and other elements, such as Cr and Ti, influence the formation of the Laves phase [23]. The atomic radii of Ti, Cr, Nb, Mo, and V are 1.32 Å, 1.46 Å, 1.25 Å, 1.43 Å, and 1.36 Å, respectively (Table 2). As the V content increases from 10 at.% to 15 at.% (AC-R1 to AC-R2), with Nb content held constant, the peak of the BCC phase shifts toward higher 2θ values. This shift is attributed to the substitution of Ti (which has a larger atomic radius) by V (which has a smaller atomic radius), leading to a reduction in the lattice parameters of the BCC phase.

After annealing, the HCP phase coarsened and partially dissolved. ANN-R5 transformed into a single-phase BCC structure, while ANN-R4 transformed into a dual-phase (BCC + HCP) structure, although the volume fraction of the HCP phase was reduced. Meanwhile, even though the precipitation of the Laves phase decreased, ANN-R1, ANN-R2, and ANN-R3 still exhibited a triple-phase (BCC + HCP + Laves) structure.

**Figure 2:**
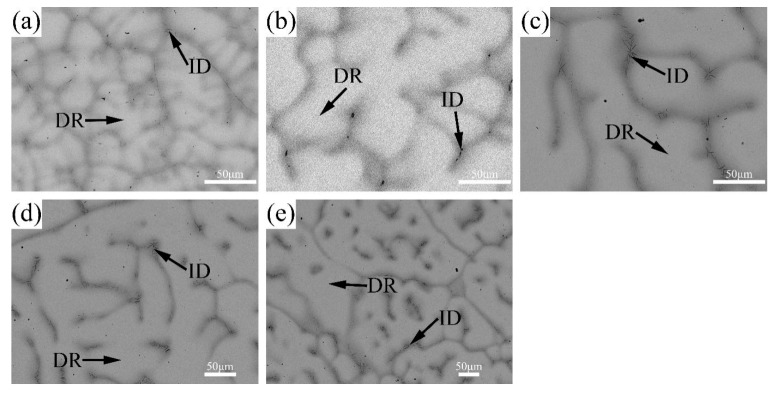
SEM-BSE images of the as-cast (**a**) AC-R1, (**b**) AC-R2, (**c**) AC-R3, (**d**) AC-R4, and (**e**) AC-R5.

**Figure 3:**
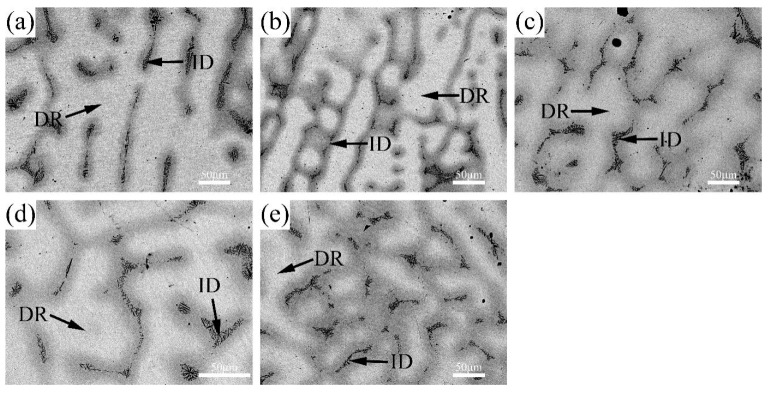
SEM-BSE images of the annealed (**a**) ANN-R1, (**b**) ANN-R2, (**c**) ANN-R3, (**d**) ANN-R4, and (**e**) ANN-R5.

**Figure 4:**
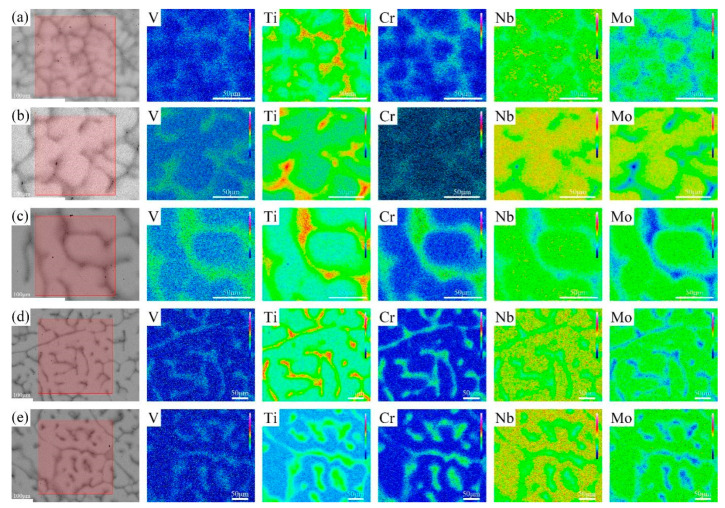
WDS maps of the elements V, Ti, Cr, Nb, and Mo, taken from the area of the backscattered electron image shown in the left panel. (**a**–**e**) are maps of the as-cast AC-R1, AC-R2, AC-R3, AC-R4, and AC-R5, respectively.

**Figure 5:**
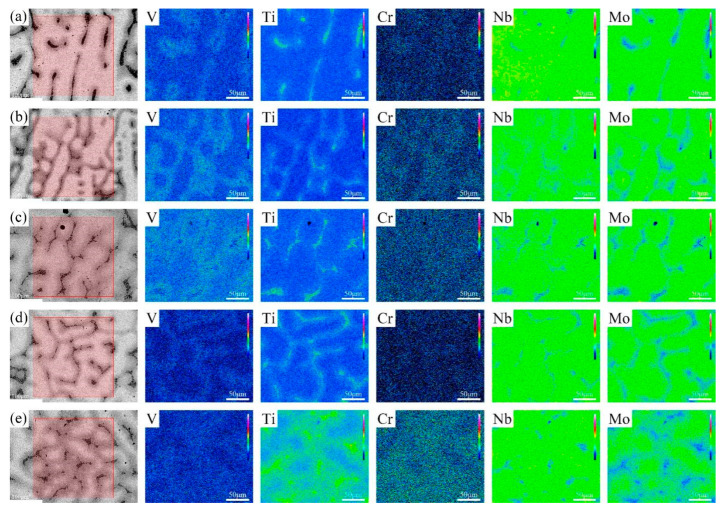
WDS maps of the elements V, Ti, Cr, Nb, and Mo, taken from the area of the backscattered electron image shown in the left panel. (**a**–**e**) are maps of the annealed ANN-R1, ANN-R2, ANN-R3, ANN-R4, and ANN-R5, respectively.

The BSE images of V-Ti-Cr-Nb-Mo RHEAs in as-cast and annealed states are shown in Figure 2 and Figure 3, respectively. A typical dendritic microstructure is observed in all alloys, attributed to differences in the melting points of the constituent elements. Such dendritic structures are commonly observed in high-entropy alloys fabricated via arc melting [14,26,27]. According to the WDS elemental mapping in Figure 4 and Figure 5, the dendritic region, which exhibits bright contrast in the BSE image, is enriched in Nb and Mo but depleted in Ti, Cr, and V. In contrast, the interdendritic region, which appears darker, is enriched in Ti, Cr, and V but depleted in Nb and Mo. During solidification, Nb and Mo, which have higher melting points, tend to precipitate first into the dendritic region. At the same time, the lower-melting-point elements Ti, Cr, and V are rejected into the interdendritic region. This segregation behavior primarily accounts for dendrite formation. Nb and Mo act as BCC phase stabilizers [28,29], while Cr and Nb promote the formation of the Laves phase [20,30], and local enrichment of Ti facilitates the formation of the HCP phase [31]. Therefore, it is inferred that the BCC phase primarily forms in the dendritic region (DR). In contrast, the Laves phase and Ti-enriched HCP phase are concentrated in the interdendritic region (ID), which is supported by subsequent TEM analysis. After annealing, atomic diffusion is enhanced due to high-temperature heat treatment, resulting in the homogenization of Ti and Cr, reduced Laves phase precipitation, dissolution of the HCP phase, and grain growth.

**Figure 6:**
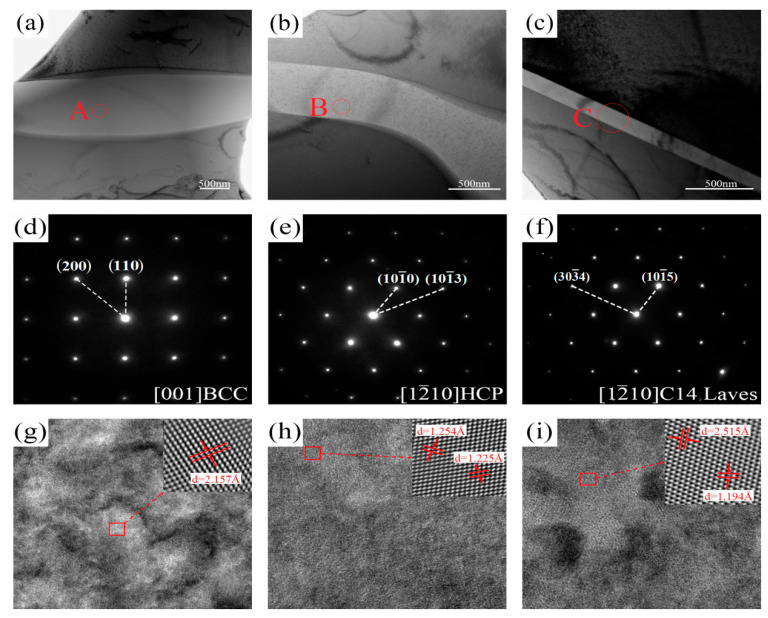
TEM results of the as-cast AC-R2. (**a**–**c**) TEM bright-field image. (**d**–**f**) The selected area diffraction pattern of region A in (**a**), B in (**b**), and C in (**c**), respectively. (**g**–**i**) HRTEM images of BCC, HCP, and C14 Laves phases.

The microstructure of AC-R2 was examined using TEM to investigate the phase structure characteristics further. Figure 6 presents the TEM bright-field (BF) image and the selected area electron diffraction patterns (SAEDPs) of AC-R2. The results show that the Nb-rich region of AC-R2 corresponds to a BCC solid solution, while the Ti-rich region comprises both HCP solid and C14 Laves solutions. Among them, the SAEDP of the C14 Laves phase is along the [1$\bar{2}$10] Laves zone axis with a lattice constant of about a = 5.03 Å, c = 8.39 Å. It can be noted that the value of c is relatively large, which may be because the lattice distortion caused by the difference in the atomic sizes in high-entropy alloys leads to a more pronounced tension strain in the direction of the c-axis [32]. In addition, the Laves phase is not a single-component intermetallic compound but rather a multi-component, complicated intermetallic compound. The TiCr₂ and NbCr₂ Laves phases coexist and belong to the space lattice group of P6_3_/mmc (194). The SAEDP of the BCC phase is along the [001] BCC zone axis, and the lattice constant of the BCC phase is about 3.05 Å. The SAEDP of the HCP phase is along the [1$\bar{2}$10] HCP zone axis, and the lattice constant of the HCP phase is about a = 2.83 Å, C = 4.38 Å.

### 3.2. Mechanical Properties

**Figure 7:**
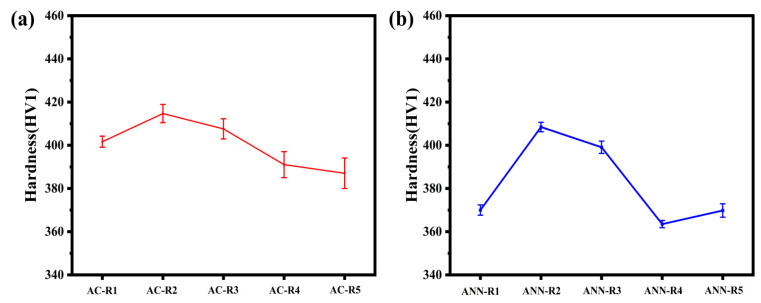
The variation in Vickers hardness of V-Ti-Cr-Nb-Mo RHEAs: (**a**) the cast; (**b**) after annealing at 1200 °C for 8 h.

**Figure 8:**
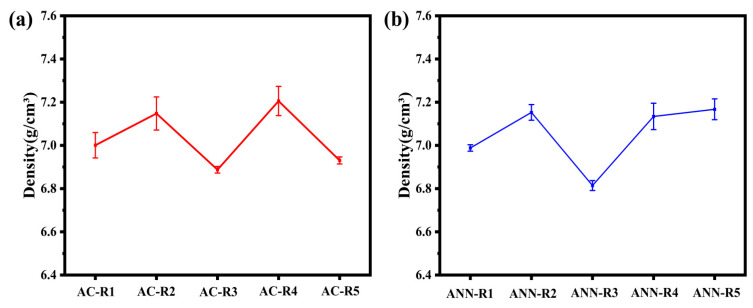
The variation in density of V-Ti-Cr-Nb-Mo RHEAs: (**a**) the cast; (**b**) after annealing at 1200 °C for 8 h.

**Figure 9:**
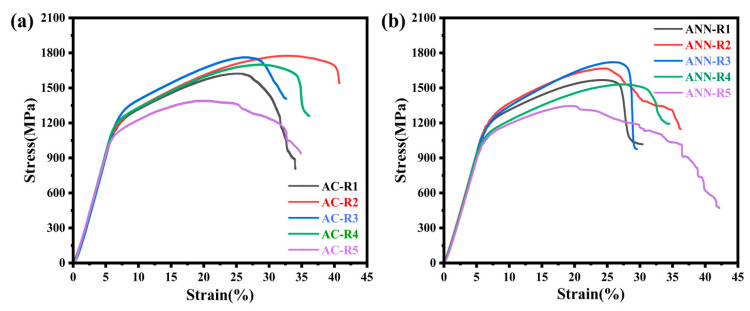
Compressive engineering stress–strain curves of V-Ti-Cr-Nb-Mo RHEAs: (**a**) the cast; (**b**) after annealing at 1200 °C for 8 h.

To investigate the intrinsic correlation between mechanical properties, alloy composition, and phase composition, hardness and density measurements were conducted, with the results presented in Figure 7 and Figure 8. Additionally, compression tests were performed. The engineering compressive stress–strain curves for the as-cast and annealed V-Ti-Cr-Nb-Mo RHEAs at room temperature are displayed in Figure 9. A comprehensive overview of the corresponding mechanical properties is provided in Table 3.

As shown in Figure 7a, the Vickers hardness values of AC-R1, AC-R2, AC-R3, and AC-R4, which contain (BCC + HCP + Laves) phases, range from 391 HV1 to 414.7 HV1. The Vickers hardness values of AC-R5, which exhibit dual-phase (BCC + HCP), are 387 HV1. These results indicate that the Laves phase is a primary strengthening phase, and an increase in its volume fraction directly contributes to an enhancement in hardness [13,33]. This conclusion is further supported by the data in Figure 7b. The Vickers hardness values of ANN-R1, ANN-R2, and ANN-R3, also containing (BCC + HCP + Laves) phases, are 370.7 HV1, 404.1 HV1, and 399.9 HV1, respectively, which are notably higher than those of ANN-R4 and ANN-R5. It is important to note that the hardness of the annealed RHEAs is significantly lower than that of the as-cast RHEAs. This reduction is attributed to two primary factors: (1) the homogeneity of the alloying constituents and the grain growth during annealing diminish the effects of solid solution strengthening and grain boundary strengthening, and (2) high-temperature annealing results in coarsening and partial dissolution of the HCP phase and reduction in precipitation of the Laves phase, thereby reducing hardness [34,35].

When the Nb content is fixed at 35 at.%, the theoretical density of the alloy is expected to increase with increasing V content. As shown in Figure 8a, the densities of AC-R5, AC-R1, and AC-R2 are 6.89 g/cm^3^, 6.96 g/cm^3^, and 7.31 g/cm^3^, respectively, which aligns well with the theoretical prediction. Notably, the densities of ANN-R1, ANN-R4, and ANN-R5 are higher than those of the corresponding as-cast samples. This increase may be attributed to the annihilation of dislocations during annealing via thermal activation, leading to denser atomic packing and, consequently, an increase in alloy density [36,37]. Previous studies have indicated that the density of Ni-based superalloys ranges from 7.6 to 9.1 g/cm^3^, while that of Co-based superalloys varies between 8.3 and 9.4 g/cm^3^ [38]. In contrast, the density of the V-Ti-Cr-Nb-Mo RHEAs designed in this study ranges from 6.81 to 7.31 g/cm^3^, significantly lower than that of traditional Ni-based and Co-based superalloys. This considerable weight reduction enhances the lightweight advantages of RHEAs, aligning with the aerospace sector’s demand for both material weight reduction and high performance.

The combination of high strength and good ductility in RHEAs has attracted significant attention in materials research and development. The single-phase BCC VTiCrNbMo RHEAs reported by C. Xiang et al. [39] exhibit a compressive strength of 1677 MPa but a relatively low ductility of only 9.4%. As shown in Figure 9 and Table 3, the ANN-R4 with a dual-phase structure of (BCC + HCP) achieves a ductility of 26.9% while maintaining a compressive strength of 1530 MPa. At comparable strength levels, the ductility of ANN-R4 increases by 186%, and the introduction of the HCP phase can effectively break through the plastic bottleneck of the traditional single-phase BCC RHEAs [40]. In addition, AC-R2, featuring a triple-phase structure of (BCC + HCP + Laves), demonstrates outstanding mechanical properties, with a compressive strength reaching 1775 MPa and ductility of 18.2%. Compared with AC-R5, which possesses dual-phase (BCC + HCP) structures, AC-R2 shows significantly enhanced strength but reduced ductility. This trade-off can be attributed to the presence of fine Laves phases within the (BCC + HCP) matrix, which hinders dislocation motion, promotes dislocation accumulation and expansion, and thereby markedly enhances the strength of RHEAs. During plastic deformation, high-density dislocation cells form near the Laves phase interfaces, and their interaction with the Laves phase effectively improves the alloy’s resistance to deformation [41]. After annealing, ANN-R2 retains the triple-phase (BCC + HCP + Laves) structure. Although its compressive strength decreases to 1665 MPa, the ductility increases substantially to 23.7%. A similar trend is observed in ANN-R1, ANN-R3, ANN-R4, and ANN-R5, where annealing leads to a reduction in strength accompanied by a significant improvement in ductility. This phenomenon can be explained by the Hall–Petch equation [42]:

$$\sigma_{gb}=\sigma_{0}+B/d^{1/2}$$

where *σ*_0_ denotes the friction resistance, and B and *d* represent the strengthening coefficient and grain diameter, respectively. Grain coarsening reduces the density of grain boundaries, thereby diminishing their ability to impede dislocation motion and leading to a decrease in strength. However, enhancing grain boundary sliding and dislocation activity facilitates the release of localized strain concentrations, thus improving ductility [43,44]. In addition, this phenomenon can also be attributed to the weakening of solid solution strengthening, the reduction in lattice distortion, and the reduction in the Laves phase precipitation after annealing. These microstructure changes effectively improve the local stress concentration phenomenon during plastic deformation, providing a more uniform deformation environment, which leads to the decline of strength and the improvement of plasticity [13,45]. As shown clearly in Figure 4 and Figure 5, annealing significantly alleviates elemental segregation. This homogeneity reduces the internal stress concentration and suppresses the formation of brittle phases, thereby further enhancing the alloy’s ductility.

**Figure 10:**
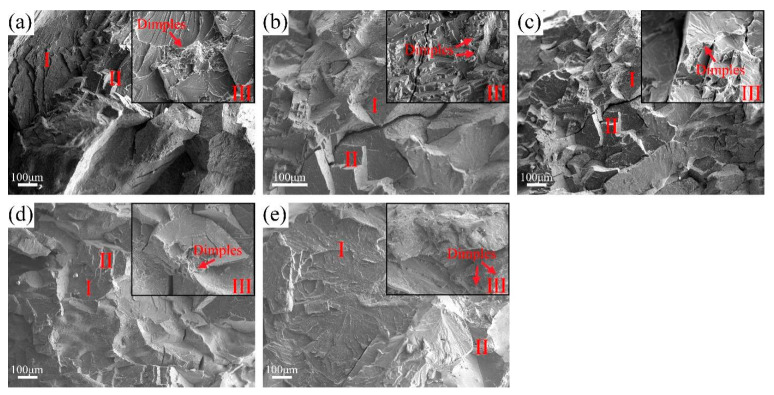
The morphologies of the fractured surface: (**a**) AC-R1, (**b**) AC-R2, (**c**) AC-R3, (**d**) AC-R4, and (**e**) AC-R5.

**Figure 11:**
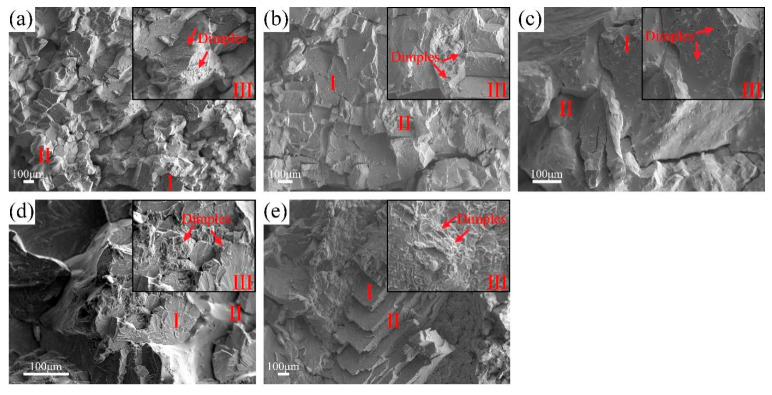
The morphologies of the fractured surface: (**a**) ANN-R1, (**b**) ANN-R2, (**c**) ANN-R3, (**d**) ANN-R4, and (**e**) ANN-R5.

Figure 10 shows the fracture morphology of AC-R1, AC-R2, AC-R3, AC-R4, and AC-R5 after compression deformation. From the fracture characteristics, it can be seen that these alloys show a mixed fracture mode of quasi-cleavage fracture (Zone Ⅰ), intergranular fracture (Zone Ⅱ), and ductile fracture (Zone Ⅲ). Among them, cleavage steps and river patterns are visible in Zone Ⅰ, indicating that crystal orientation and crack propagation path greatly affect the fracture process in local areas. The fracture morphology of the annealed samples of the five alloys is shown in Figure 11, which also shows the synergy of three fracture mechanisms, namely the mixed mode of quasi-cleavage fracture (Zone Ⅰ), intergranular fracture (Zone Ⅱ), and ductile fracture (Zone Ⅲ). Compared with the as-cast sample, denser dimples are formed on the fracture surface of the annealed sample. In particular, it can be observed in Figure 11d,e that the dimple size increases significantly and the depth also increases, reflecting the significant increase in the ductility level of RHEAs after annealing, which is consistent with the analysis results of the compression experiment.

**Figure 12:**
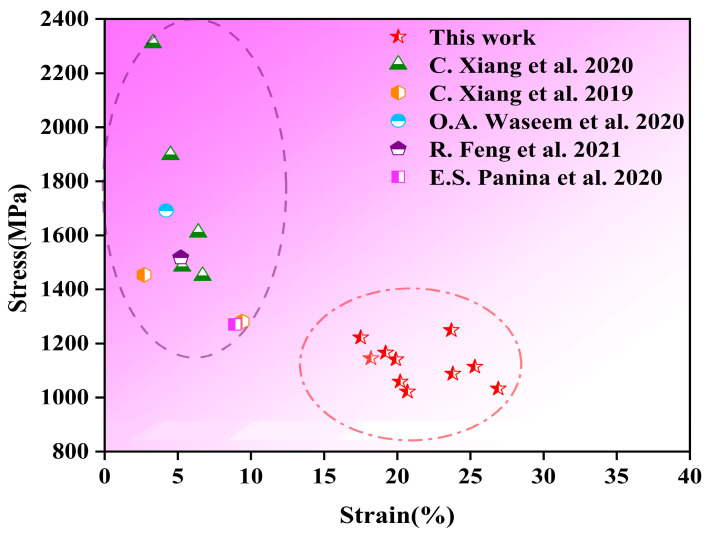
Figures of the compressive yield strength and strain of the V-Ti-Cr-Nb-Mo RHEAs in this work and several typical RHEAs in the previously mentioned studies at room temperature: C. Xiang et al. [20,39], R. Feng et al. [47], O.A. Waseem et al. [46], and E.S. Panina et al. [48].

In general, as shown in Figure 12, the yield strength of V-Ti-Cr-Nb-Mo RHEAs ranges from 1022 MPa to 1222 MPa, with strains between 17.5% and 26.9%. Compared with other representative RHEAs reported in the literature, the ductility substantially increases by 86–896% [20,39,46,47,48]. As presented in Table 4, both the as-cast and annealed V-Ti-Cr-Nb-Mo RHEAs exhibit a favorable strength–ductility synergy attributed to their multi-phase structures. Specifically, due to their inherent ductility, the BCC and HCP phases often serve as matrix or ductile phases. In contrast, the Laves phase, characterized by its high hardness and brittleness, typically acts as a strengthening phase that contributes to the overall enhancement of mechanical strength.

## 4. Conclusions

In this study, five kinds of V-Ti-Cr-Nb-Mo RHEAs were designed and prepared by arc melting. The effects of varying proportions of Ti, V, and Nb, along with annealing treatments, on the crystal structure, microstructural evolution, and mechanical properties of the RHEAs were systematically investigated, providing an experimental basis for optimizing alloy composition and performance control. The main conclusions follow.

The strategy of controlling a multi-phase approach by adjusting elemental ratios and heat treatments proved effective. The five as-cast RHEAs exhibited a dual-phase (BCC + HCP) structure, while AC-R1, AC-R2, AC-R3, and AC-R4 achieved coordinated control of a triple-phase (BCC + HCP + Laves) structure. The results indicate that the prepared RHEAs possess a dendritic microstructure, with Nb and Mo enriched in the dendritic regions and Ti, Cr, and V concentrated in the interdendritic areas. When the Nb content is maintained at 35 at.%, and the V content is increased, the BCC phase’s lattice parameters decrease, the HCP phase’s volume fraction increases, and the C14 Laves phase emerges. Following heat treatment, grain growth occurs, elemental segregation is significantly reduced, the HCP phase undergoes coarsening and partial dissolution, and the Laves phase precipitation decreases. The ANN-R1, ANN-R2, and ANN-R3 retain the coordinated regulation of a triple-phase (BCC + HCP + Laves) structure, while ANN-R4 transforms into a dual-phase (BCC + HCP) structure.

Multi-phase RHEAs can enhance the synergy between strength and ductility. The ANN-R4 with a dual-phase (BCC + HCP) structure exhibits a ductility of 26.9% under a compressive strength of 1530 MPa. The AC-R2, featuring a triple-phase (BCC + HCP + Laves) structure, achieves a compressive strength of 1775 MPa and a ductility of 18.2%. Compared with previously reported V-Ti-Cr-Nb-Mo RHEAs in the literature, the RHEAs developed in this study exhibit significantly enhanced ductility (increased by 86–896%) while maintaining high compressive yield strength (ranging from 1022 MPa to 1222 MPa). It can be seen that introducing a multi-phase structure into RHEAs is an effective method to improve mechanical properties.

## Figures and Tables

**Table 1:** Compositions of the designed RHEAs and the corresponding composition code (at.%).

As-Cast Composition Code	Composition (at.%)
AC-R1	10V-35Ti-5Cr-35Nb-15Mo
AC-R2	15V-30Ti-5Cr-35Nb-15Mo
AC-R3	15V-35Ti-5Cr-30Nb-15Mo
AC-R4	5V-35Ti-5Cr-40Nb-15Mo
AC-R5	5V-40Ti-5Cr-35Nb-15Mo
**Annealed Composition Code**	**Composition (at.%)**
ANN-R1	10V-35Ti-5Cr-35Nb-15Mo
ANN-R2	15V-30Ti-5Cr-35Nb-15Mo
ANN-R3	15V-35Ti-5Cr-30Nb-15Mo
ANN-R4	5V-35Ti-5Cr-40Nb-15Mo
ANN-R5	5V-40Ti-5Cr-35Nb-15Mo

**Table 2:** Atomic radius (r), melting point (T_m_), and density ρ (g/cm^3^) of the pure Mo, V, Nb, Ti, and Cr elements. [9,24,25].

Element	V	Ti	Cr	Nb	Mo
ρ, g/cm^3^	6.11	4.51	7.14	8.57	10.28
r, Å	1.32	1.46	1.25	1.43	1.36
T_m_, (°C)	1910	1668	1907	2477	2623

**Table 3:** Mechanical properties of V-Ti-Cr-Nb-Mo RHEAs at room temperature, compressive yield strength σ_0.2_ (MPa), ultimate compressive strength σ_b_ (MPa), strain ε (%), hardness (HV1), and density ρ (g/cm^3^).

Alloy	σ_0.2_ (MPa)	σ_b_ (MPa)	ε (%)	Hardness (HV1)	ρ (g/cm^3^)
AC-R1	1141	1622	19.9	401.7 ± 2.57	6.96 ± 0.07
AC-R2	1145	1775	18.2	414.7 ± 4.21	7.31 ± 0.26
AC-R3	1222	1759	17.5	407.6 ± 4.68	6.95 ± 0.11
AC-R4	1166	1701	19.2	391.0 ± 6.01	7.08 ± 0.21
AC-R5	1059	1388	20.2	387.0 ± 7.04	6.89 ± 0.12
ANN-R1	1088	1567	23.8	370.7 ± 3.21	6.99 ± 0.01
ANN-R2	1098	1665	23.7	404.1 ± 7.73	7.15 ± 0.04
ANN-R3	1114	1720	25.3	399.9 ± 6.47	6.81 ± 0.02
ANN-R4	1033	1530	26.9	363.8 ± 2.51	7.13 ± 0.06
ANN-R5	1022	1344	20.7	367.9 ± 7.82	7.17 ± 0.05

**Table 4:** The phase composition, state, ultimate compressive strength, and strain of the V-Ti-Cr-Nb-Mo RHEAs in this work, along with several typical V-Ti-Cr-Nb-Mo RHEAs from previous studies by C. Xiang et al. [20,39] at room temperature.

Alloy	Phase Composition	State	σ_b_ (MPa)	ε (%)	References
AC-R2	BCC + HCP + Laves	As-cast	1775	18.2	This work
AC-R3	BCC + HCP + Laves	As-cast	1759	17.5	This work
AC-R4	BCC + HCP + Laves	As-cast	1701	19.2	This work
AC-R1	BCC + HCP + Laves	As-cast	1622	19.9	This work
AC-R5	BCC + HCP	As-cast	1388	20.2	This work
VTiCrNbMo	BCC	As-cast	1677	9.4	[39]
ANN-R2	BCC + HCP + Laves	AT-1200 °C	1665	23.7	This work
ANN-R3	BCC + HCP + Laves	AT-1200 °C	1720	25.3	This work
ANN-R1	BCC + HCP + Laves	AT-1200 °C	1567	23.8	This work
ANN-R4	BCC + HCP	AT-1200 °C	1530	26.9	This work
VTiCr_0.75_NbMo_0.5_	BCC	AT-1200 °C	1543	5.3	[20]
VTiCr_0.5_NbMo_0.5_	BCC	AT-1200 °C	1586	6.7	[20]

## Data Availability

The original contributions presented in the study are included in the article, further inquiries can be directed to the corresponding authors.
